# Deletion of Lymphatic PD‐L1 Protects Mice From Severe Autoimmune Encephalitis

**DOI:** 10.1002/eji.70175

**Published:** 2026-03-29

**Authors:** Ziyuan Wang, Elèni Meuffels, Christian Ashworth, Emma Reynaud, Shruthi Hemanna, Burkhard Becher, Michael Detmar, Sarah Mundt, Lothar C. Dieterich

**Affiliations:** ^1^ European Center for Angioscience (ECAS) Medical Faculty Mannheim Heidelberg University Heidelberg Germany; ^2^ Mannheim Institute of Innate Immunoscience (MI3) Medical Faculty Mannheim Heidelberg University Heidelberg Germany; ^3^ Heidelberg Bioscience International Graduate School (HBIGS) Faculty of Biosciences Heidelberg University Heidelberg Germany; ^4^ Institute of Experimental Immunology University of Zurich Zurich Switzerland; ^5^ Institute of Pharmaceutical Sciences Swiss Federal Institute of Technology (ETH) Zurich Zurich Switzerland; ^6^ Department of Neurology University Hospital Zurich Zurich Switzerland

## Abstract

Lymphatic endothelial cells at the cribriform plate and in brain‐draining cervical lymph nodes express PD‐L1 during autoimmune encephalitis. Unexpectedly, deletion of lymphatic PD‐L1 expression ameliorates disease symptoms and is associated with increased brain infiltration of regulatory CD8+ Foxp3+ T cells.

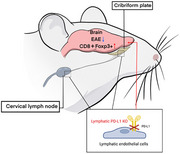

The lymphatic vascular system is crucial for immune regulation, not only through transport of antigen and immune cells to and from lymph nodes (LNs) but also through direct immune‐regulatory functions of lymphatic endothelial cells (LECs) [1]. Remarkably, LN‐resident LECs constitutively express programmed death ligand 1 (PD‐L1), particularly in the floor of the subcapsular and in medullary sinuses [2]. In addition, peripheral LECs upregulate PD‐L1 expression in the context of inflammation and cancer [1], suggesting that they may directly be involved in immunoregulation. Congruently, we previously found that mice lacking PD‐L1 expression in LECs (PD‐L1^LECKO^) show elevated CD8^+^ T‐cell responses against tumor antigens [3]. However, whether lymphatic PD‐L1 also controls T‐cell‐mediated autoimmunity is unknown.

Previously considered an immune‐privileged site, work over the past decade has demonstrated that the central nervous system (CNS) is drained by lymphatic vessels [4]. Lymphatic vessels associated with the cribriform plate and connected to the dense lymphatic network in the nasal mucosa account for a major fraction of the CNS drainage [5]. Interestingly, cribriform plate–associated LECs strongly upregulate PD‐L1 expression during experimental autoimmune encephalitis (EAE), a mouse model of multiple sclerosis (MS) [6]. However, it is currently unknown whether lymphatic PD‐L1 expression is relevant for EAE severity in vivo.

To address this question, we challenged PD‐L1^LECKO^ mice [3], in which PD‐L1 is inducibly deleted upon tamoxifen treatment in Prox1+ LECs (Figure S1), with EAE. After immunization with an MHC‐II‐restricted peptide derived from the self‐antigen myelin oligodendrocyte glycoprotein (MOG), both PD‐L1^LECKO^ (Cre+) and littermate controls (Cre−) developed typical symptoms of EAE. Unexpectedly, we found that the clinical EAE scores were significantly reduced in Cre+ mice (Figure 1A), resulting in a trend toward longer survival (Figure 1B) and a significantly reduced rate of mice developing severe symptoms (score ≥ 3) in the Cre+ group (Figure 1C).

**FIGURE 1:**
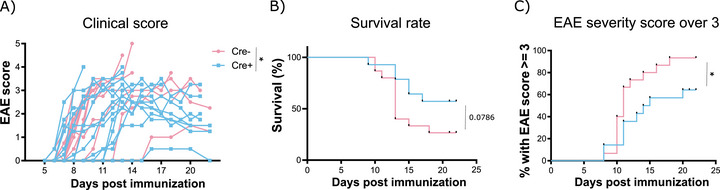
Lymphatic PD‐L1 deletion protects from severe EAE. (A) Cre− (*N* = 15) and Cre+ mice (*N* = 14) were treated with tamoxifen and subsequently immunized with MOG_35‐55_. Clinical symptoms were scored over time, and mice were euthanized when a score of 4 was reached. * *p* < 0.05 (two‐way ANOVA test). (B) Kaplan–Meier survival curve of the same mice as in (A). (C) Proportion of mice from (A) developing severe EAE (score 3 or higher). * *p* < 0.05 (log‐rank test).

To dissect the impact of lymphatic PD‐L1 deletion on the T‐cell landscape in EAE, we performed deep immunophenotyping of LNs and the CNS by high‐dimensional flow cytometry. First, we analyzed peripheral LNs draining the immunization site prior to disease onset (day 6), but found no major differences between Cre+ and Cre− mice, neither with regard to CD8+ and CD4+ T‐cell frequencies nor their phenotype (Figure S2).

As the rate of developing severe EAE appeared attenuated in Cre+ mice especially from day 10 after immunization and onward (Figure 1C), we next assessed the CNS T‐cell landscape in Cre+ and Cre− mice on day 13 after immunization (Figure 2A). While the frequency of CNS‐infiltrating CD45+ leukocytes as well as TCRb+ CD4+ and CD8+ T cells remained unaltered (Figure 2B,C, Figure S3A), we detected a small subset of CD8+ Foxp3+ cells, which selectively emerged in Cre+ mice lacking lymphatic PD‐L1 (Figure 2D,E). Of note, most of these cells also expressed Helios (Figure 2D), a transcription factor associated with Treg stability [7]. In line with previous descriptions [8], CD8+ FoxP3+ cells expressed higher levels of CD25, CD103, CTLA4, and GITR compared to CD8+ Foxp3− cells on their surface (Figure 2F,G). These cells also robustly expressed PD‐1, although at a lower level than their Foxp3− counterparts (Figure 2F,G). Closer inspection furthermore demonstrated an increased proliferation rate (evidenced by Ki67 staining) and upregulation of CTLA4 by CD8+ Foxp3+ cells in Cre+ compared to Cre− mice (Figure 2G,H). In contrast, canonical CD4+ Foxp3+ T regs in the inflamed CNS appeared largely unaffected by lymphatic PD‐L1 deletion (Figure S3B–F). Mapping the T‐cell responses in the cervical, brain‐draining LNs at peak disease also revealed no major changes between Cre− and Cre+ animals (Figure S4).

**FIGURE 2:**
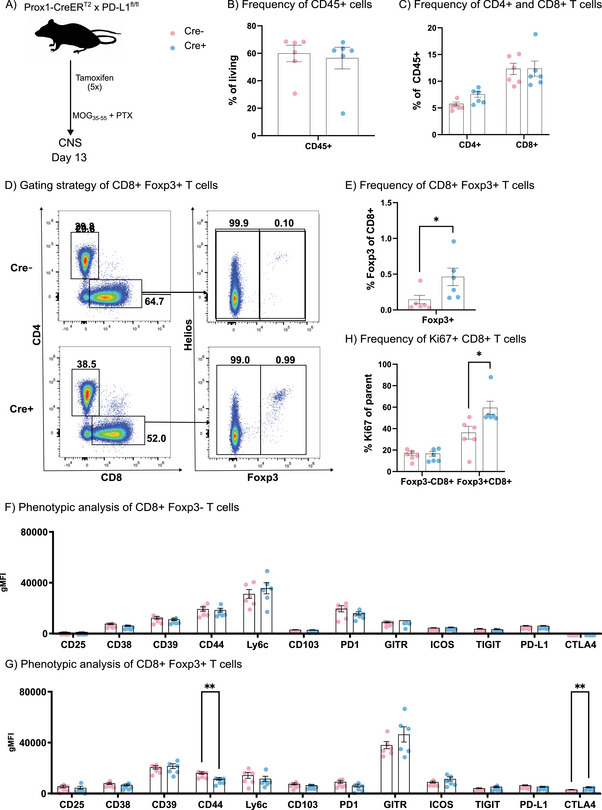
Lymphatic PD‐L1 depletion amplifies regulatory CD8+ T cells in the inflamed CNS. (A) Schematic of the experimental approach. (B) Frequency of CD45+ cells infiltrating the CNS. (C) Frequency of TCRb+ CD4+ and TCRb+ CD8+ T cells in the CNS. (D) Representative FACS plots of CNS‐infiltrating T cells (pre‐gated living singlet TCRb+ cells, see Figure S3A) gated for CD4 versus CD8 and subsequently examined for Foxp3 and Helios expression. (F, G) Phenotypic analysis of CD8+ Foxp3− T cells (F) and CD8+ Foxp3+ T cells (G) expressed as geometric mean intensity for the molecules indicated on the *x*‐axis. (H) Frequency of Ki67+ cells among CD8+ Foxp3− and CD8+ Foxp3+ T cells in the CNS on day 13. Bar graphs indicate mean ± SEM. *N* = 6 mice/group. * *p* < 0.05, ** *p* < 0.01, Student's *t*‐test.

Taken together, our data indicate that lymphatic PD‐L1 deletion alleviates clinical symptoms in an EAE mouse model, which may be causally linked to a selective unleashing of CD8+ Tregs with suppressive capacities. At first glance, these findings appear to be at odds with the well‐known immune‐inhibitory role of PD‐L1. However, the disease context and contribution of distinct T‐cell subsets to disease pathology deserve consideration. Recent reports have uncovered a regulatory function of CD8^+^ T cells in EAE [9]. Furthermore, CD8+ Foxp3+ T cells were found to be decreased in the blood of relapsing MS patients, suggesting a potential role of these cells in autoimmune neuroinflammation [10]. Consequently, we suggest that in the context of EAE, lymphatic PD‐L1 deletion unleashes a CD8+ T reg population that infiltrates the CNS and ameliorates neuroinflammation.

## Conflicts of Interest

The authors declare no conflicts of interest.

## Supporting information




**Supporting File 1**: eji70175‐sup‐0001‐SupMat.pdf.

## Data Availability

Flow cytometry raw data that support the findings of this study are available from the corresponding author upon reasonable request.
