# Supplementary material for: Deletion of Lymphatic PD‐L1 Protects Mice From Severe Autoimmune Encephalitis
Source: Eur J Immunol. 2026 Mar 29;56(4):e70175. doi: 10.1002/eji.70175 (PMC13033968; doi:10.1002/eji.70175)
Supplement: Supplementary file 1 — Supporting File 1: eji70175‐sup‐0001‐SupMat.pdf. [file EJI-56-e70175-s001.pdf]

Wang, Meuffels et al.

Deletion of lymphatic PD-L1 protects mice from severe autoimmune encephalitis

Material and Methods

Mice: PD-L1^{LECKO} mice (Prox1-Cre-ER^{T2} x Cd274$^{fl/fl}$) on a C57Bl/6 background have been described previously (*1*) and were bred under SOPF conditions. To induce Cre-mediated recombination, 50 mg/kg tamoxifen (Sigma) in sunflower oil (Sigma) were administered by i.p. injection for 5 days. Cre-negative Cd274$^{fl/fl}$ littermates served as controls and were equally treated with tamoxifen. All animal procedures were reviewed and approved by the Swiss Veterinary Office and performed according to institutional and federal guidelines.

Experimental autoimmune encephalitis (EAE) model: To elicit neuroinflammation, sex matched, adult male and female mice between 8-12 weeks of age, were immunized with myelin oligodendrocyte glycoprotein (MOG) peptide as described previously (*2*). In brief, 200 µg of MOG$_{35-55}$ peptide (Genscript) emulsified in complete Freund's adjuvant (BD) were injected s.c. and 100 ng pertussis toxin (List Biological Laboratories, 179A) was injected i.p. on the day of immunization and again 2 days after. Scoring of EAE symptoms was done as follows: 0: no signs of EAE; 0.5: tail limp at distal end; 1: entire limp tail; 1.5: limp tail and hind limb weakness; 2: unilateral partial hind limb paralysis; 2.5: bilateral partial hind limb paralysis; 3: complete bilateral hind limb paralysis; 3.5: complete bilateral hind limb and partial forelimb paralysis; 4: complete hind and forelimb paralysis (moribund). 0.5 scoring intervals were used to allow a more accurate description of the clinical symptoms.

Tissue processing and flow cytometry: Peripheral axillary and inguinal LNs, CNS-draining superficial cervical LNs and brain were collected at the indicated timepoints and processed for flow cytometry. For leukocyte analysis, LNs and brain were digested using 0.4 mg/ml Collagenase IV (Sigma) and 0.2 mg/ml DNAse 1 (Luzerna) in HBSS for 40 min at 37°C. Subsequently, the material was mechanically dissociated using a 19-gauge needle and filtered through a 100 µm cell strainer to obtain a single cell suspension. In the case of brain samples, leukocytes were further enriched by 30% Percoll gradient centrifugation. For analysis of LN stromal cells, LN samples were digested as described previously (*3*). In brief, LN capsules were broken using needles, followed by sequential digestion with 1 mg/ml and then 3.5 mg/ml Collagenase IV (Gibco) in DMEM supplemented with 2% FBS (Gibco) and 1.2 mM $CaCl_2$. Finally, the material was passed through a 40 µm cell strainer to obtain a single cell suspension. FACS staining was done on ice using fluorescently conjugated primary antibodies (Supplementary Table 1) in combination with an Fc-blocker (rat anti-mouse CD16/32, clone 93, Biolegend). Intracellular staining was achieved using the Foxp3 staining buffer kit (eBioscience) according to the manufacturer's instructions.

Data were acquired on a 5-laser Aurora spectral analyzer (Cytek Biosciences) or a FACS Aria II (BD) and analyzed using FlowJo (BD). For the specific gating of TCRβ+ T cells, we included negative gating for MHCII, CD19, and Ly6G.

Author contributions

Experiments and data analysis: Z.W., E.M., C.A., E.R, S.H; ressources: B.B., M.D., S.M., L.C.D.; conepualization: M.D., S.M., L.C.D.; supervision: S.M., L.C.D; manuscript writing: Z.W., S.M., L.C.D.; review: Z.W., E.M., C.A., E.R., S.H., B.B., M.D., S.M., L.C.D.

References

1. N. Cousin *et al.*, Lymphatic PD-L1 expression restricts tumor-specific CD8+ T cell responses. *Cancer Res.*, (2021).
2. M. Andreadou *et al.*, IL-12 sensing in neurons induces neuroprotective CNS tissue adaptation and attenuates neuroinflammation in mice. *Nat. Neurosci.* **26**, 1701-1712 (2023).
3. C. D. Commerford *et al.*, Mechanisms of Tumor-Induced Lymphovascular Niche Formation in Draining Lymph Nodes. *Cell Rep* **25**, 3554-3563 e3554 (2018).

Supplementary Tables:

Antigen	Supplier	Clone	Dilution	Fluorchrome	Art.nr
CD8a	BD Biosciences	53-6.7	1:150	BUV 805	612898
CD44	BD Biosciences	IM7	1:400	BUV 737	612799
CD19	BD Biosciences	1D3	1:100	BUV 661	612971
Ly6G	BD Biosciences	1A8	1:200	BUV 563	612921
CD4	BD Biosciences	GK1.5	1:200	BUV 496	612952
CD45	BD Biosciences	30-F11	1:300	BUV 395	564279
CD279/PD-1	BioLegend	29F.1A12	1:300	BV 785	135225
Ly-6C	BioLegend	HK1.4	1:500	BV 711	128037
CD25	BioLegend	PC61	1:100	BV 650	102038
CD152/CTLA4	BioLegend	UC10-4B9	1:400	BV 605	106323
CD62L	BioLegend	MEL-14	1:200	BV 570	104433
KI67	BD Biosciences	B56	1:200	BV480	566109
CD278/ICOS	BioLegend	C398.4A	1:100	Pacific Blue	313521
CD103	BioLegend	2E7	1:100	BV 421	121422
CD39	eBioscience	24DMS1	1:500	PerCPefluor 710	46-0391-80
CD357/GITR	BioLegend	DTA-1	1:400	FITC	126308
CD274/PD-L1	eBioscience	MIH5	1:500	PE-Cy7	25-5982-82
TCR beta chain	BioLegend	H57-597	1:300	PE-Cy5	109210
Foxp3	eBioscience	FJK-16s	1:400	PE-eFlour610	61-5773-82
TIGIT	BioLegend	1G9	1:50	PE	142104
CD38	Biolegend	90	1:400	APC-Fire810	102746
Zombie NIR™ Fixable Viability Kit	Biolegend		1:1000	Zombie NIR	423106
I-A / I-E	BioLegend	M5/114.15.2	1:400	AlexaFlour 700	107622
HELIOS	BioLegend	22F6	1:200	AlexaFlour 647	137218

Table S1: Specifications of antibodies used for flow cytomtery

Supplementary Figures:

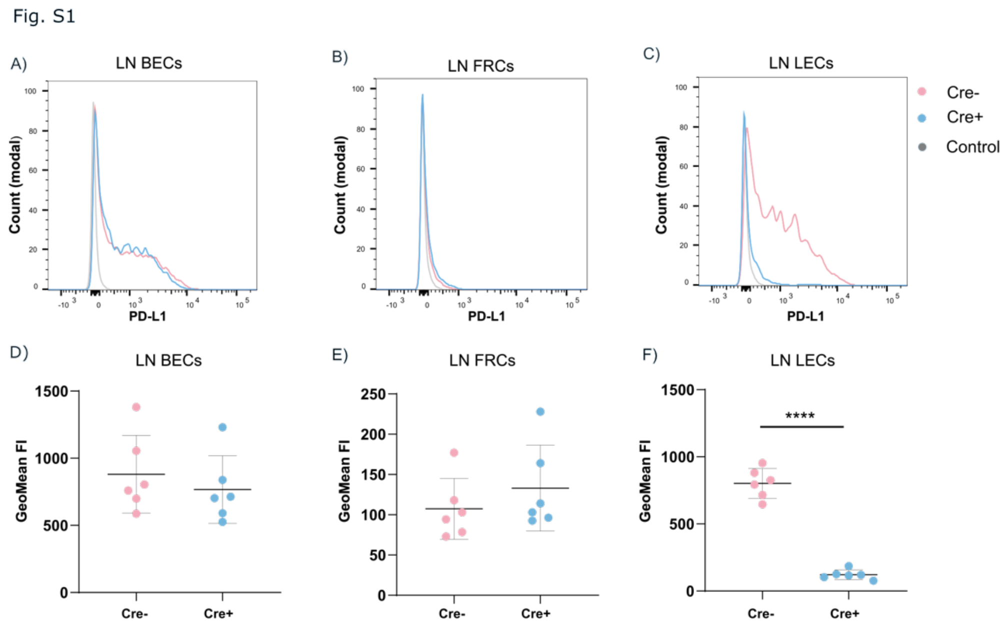

Figure S1: Flow cytometry quantification of PD-L1 expression in peripheral LN stromal cells of PD-L1^{LECKO} mice. (A-C) Representative histograms of PD-L1 expression in CD45- CD31+ Podoplanin+ blood endothelial cells (BECs), CD45- CD31- Podoplanin+ fibroblastic reticular cells (FRCs) and CD45- CD31+ Podoplanin+ lymphatic endothelial cells (LECs) after tamoxifen application and MOG vaccination. Red curve: Cre-negative; blue curve: Cre-positive; grey curve: isotype control staining. (D-F) Quantification of PD-L1 expression in peripheral LN BECs, FRCs, and LECs. Graphs represent mean ± SD. N = 6 mice / group, **** $p < 0.0001$, unpaired Student's t-test.

Fig.S2

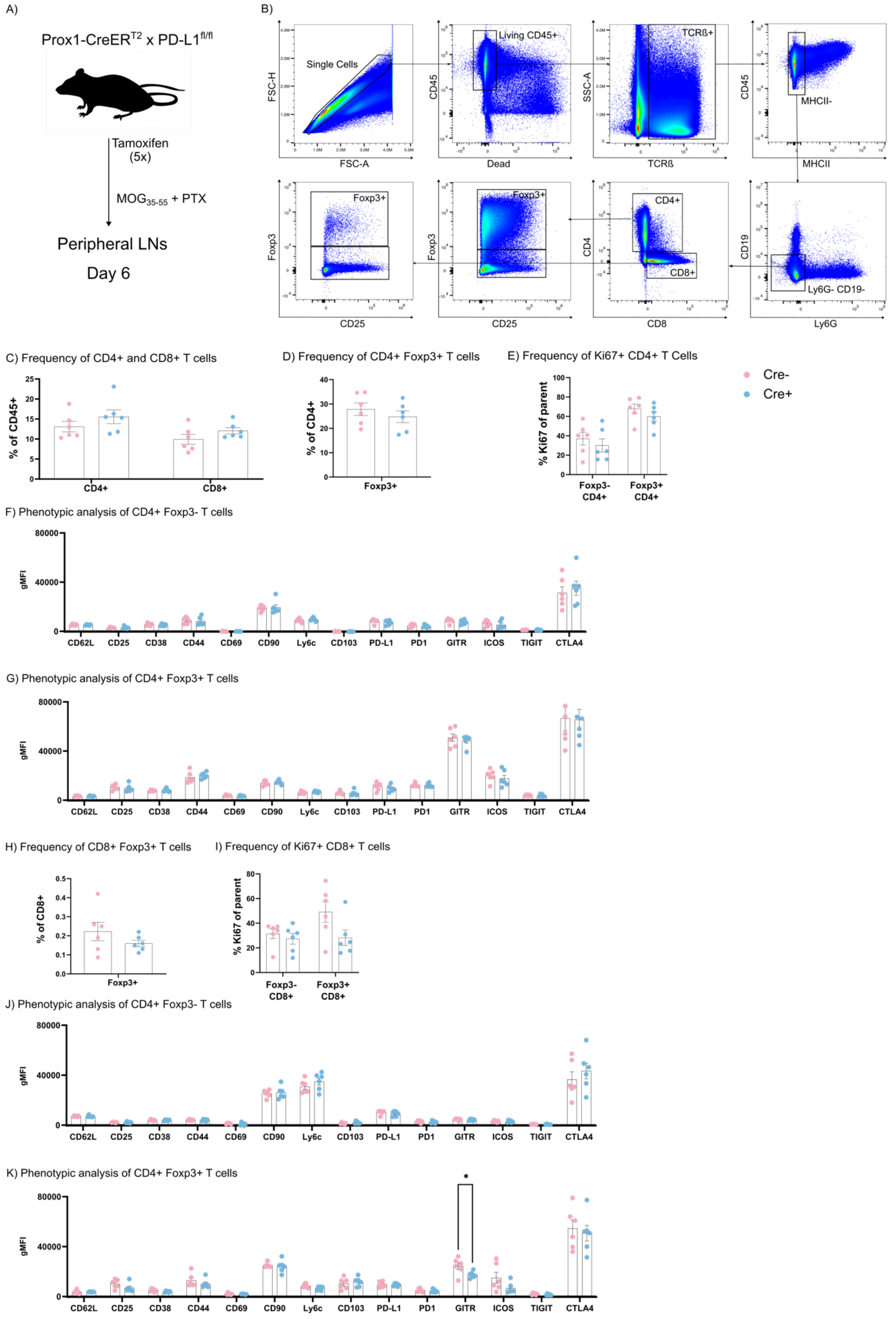

A)
Prox1-CreERT2 x PD-L1fl/fl
Tamoxifen (5x)
MOG35-55 + PTX
Peripheral LNs
Day 6
B)
Single Cells
FSC-H
FSC-A
Living CD45+
CD45
Dead
TCRß+
SSC-A
TCRß
MHCII-
MHCII
Foxp3+
Foxp3
CD25
CD4+
CD8+
CD4
CD8
Ly6G- CD19-
CD19
Ly6G
C) Frequency of CD4+ and CD8+ T cells
% of CD45+
CD4+
CD8+
D) Frequency of CD4+ Foxp3+ T cells
% of CD4+
Foxp3+
E) Frequency of Ki67+ CD4+ T Cells
% Ki67 of parent
Foxp3- CD4+
Foxp3+ CD4+
Cre-
Cre+
F) Phenotypic analysis of CD4+ Foxp3- T cells
gMFI
CD62L CD25 CD38 CD44 CD69 CD90 Ly6c CD103 PD-L1 PD1 GITR ICOS TIGIT CTLA4
G) Phenotypic analysis of CD4+ Foxp3+ T cells
gMFI
CD62L CD25 CD38 CD44 CD69 CD90 Ly6c CD103 PD-L1 PD1 GITR ICOS TIGIT CTLA4
H) Frequency of CD8+ Foxp3+ T cells
% of CD8+
Foxp3+
I) Frequency of Ki67+ CD8+ T cells
% Ki67 of parent
Foxp3- CD8+
Foxp3+ CD8+
J) Phenotypic analysis of CD4+ Foxp3- T cells
gMFI
CD62L CD25 CD38 CD44 CD69 CD90 Ly6c CD103 PD-L1 PD1 GITR ICOS TIGIT CTLA4
K) Phenotypic analysis of CD4+ Foxp3+ T cells
gMFI
*
CD62L CD25 CD38 CD44 CD69 CD90 Ly6c CD103 PD-L1 PD1 GITR ICOS TIGIT CTLA4

Figure S2: Immunophenotyping of the T cell landscape in peripheral LNs on day 6 after MOG immunization. (A) Schematic representation of the experiment. (B) Gating strategy to identify CD4+ and CD8+ T cells including their Foxp3+ subsets. Example derived from a Cre- mouse. (C) Frequency of CD4+ and CD8+ T cells in peripheral LNs draining the site of immunization. (D) Frequency of CD4+ Foxp3+ Tregs. (E) Frequency of Ki67+ cells among CD4+ Foxp3- and CD4+ Foxp3+ T cells. (F-G) Phenotypic analysis of CD4+ Foxp3- T cells (F) and CD4+ Foxp3+ T cells (G) expressed as geometric mean intensity (gMFI) for the molecules indicated. (H) Frequency of CD8+ Foxp3+ T cells. (I) Frequency of Ki67+ cells among CD8+ Foxp3- and CD8+ Foxp3+ T cells. (J-K) Phenotypic analysis of CD8+ Foxp3- T cells (J) and CD8+ Foxp3+ T cells (K) expressed as geometric mean intensity for the indicated molecules. Graphs represent mean ± SEM. N = 6 mice / group. * $p < 0.05$, Student's t-test.

Fig.S3

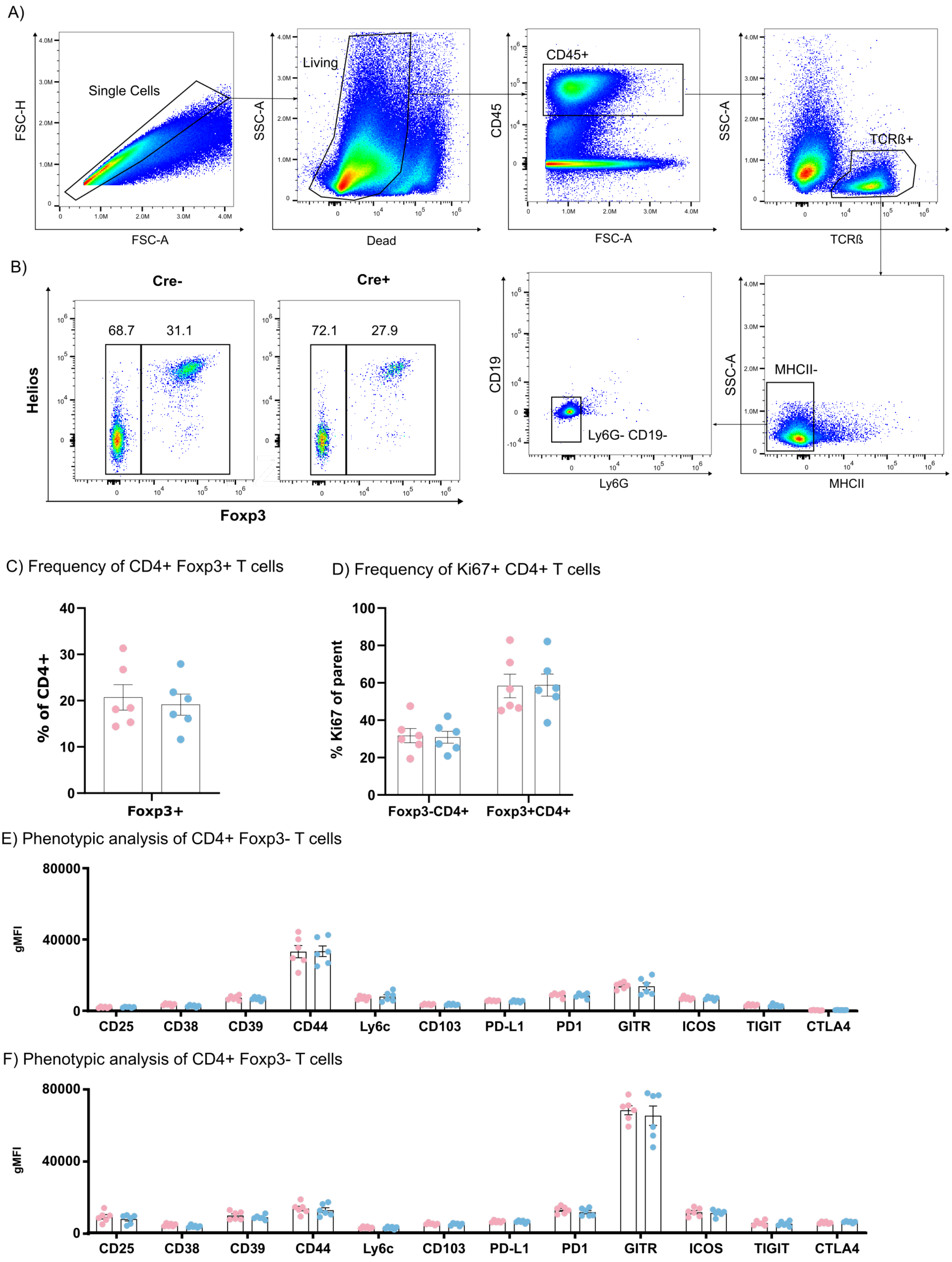

Figure S3: Characterization of CNS-infiltrating T cells on day 13 after immunization. (A) Gating strategy for TCRβ+ T cells in the CNS. The example shown is derived from a Cre-

mouse. (B) Representative FACS plots showing Foxp3 and Helios expression in CD4+ T cells in the CNS. (C) Frequency of CD4+ Foxp3+ Tregs in the CNS. (D) Frequency of Ki67+ cells among CD4+ Foxp3- and CD4+ Foxp3+ T cells in the CNS. (E-F) Phenotypic analysis of CD4+ Foxp3- T cells (E) and CD4+ Foxp3+ T cells (F) expressed as geometric mean intensity (gMFI) for the indicated molecules. Graphs represent mean ± SEM. N = 6 mice / group.

Fig.S4

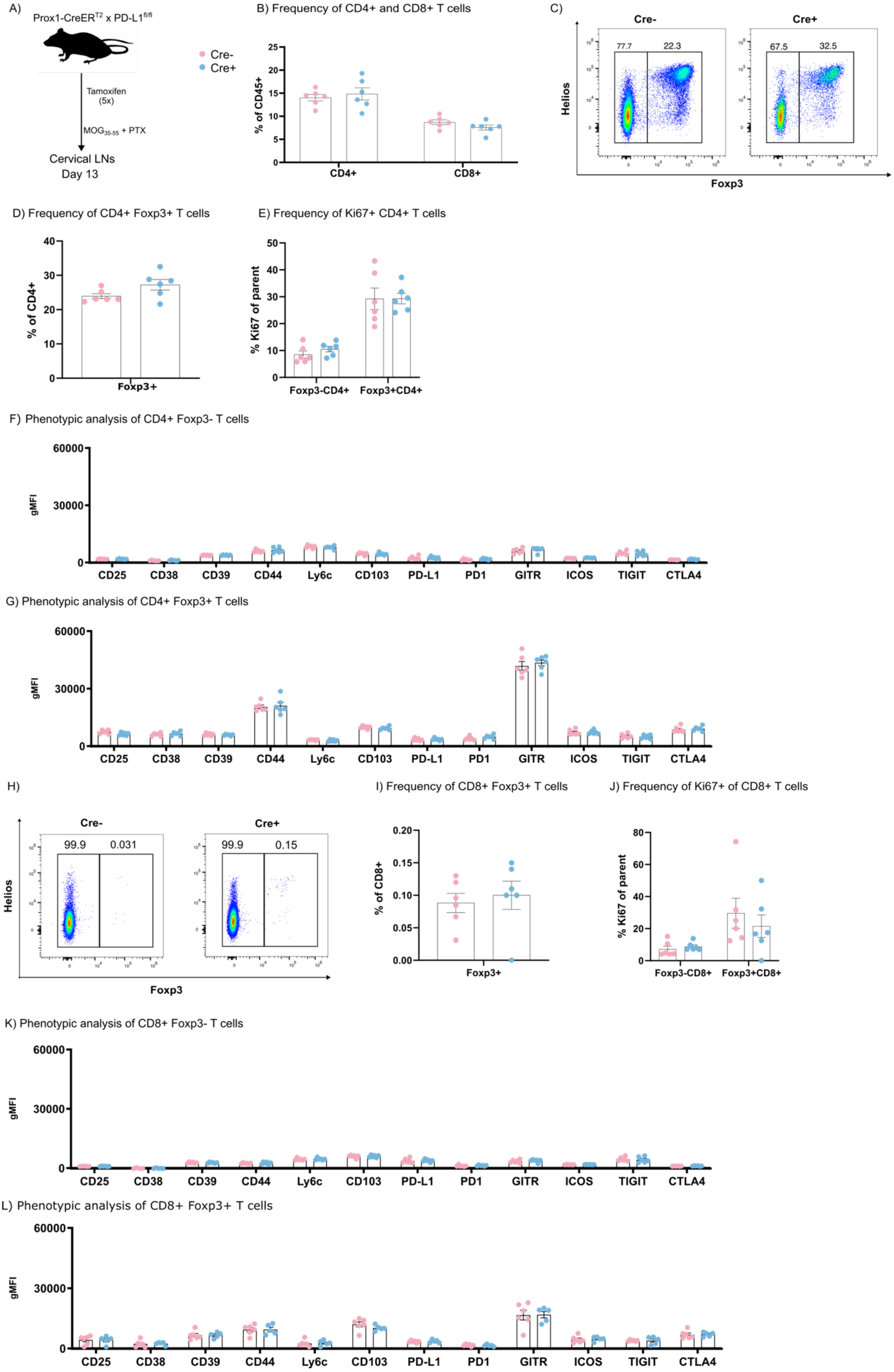
A)
Prox1-CreERT2 x PD-L1fl/fl
Tamoxifen (5x)
MOG35-55 + PTX
Cervical LNs
Day 13
Cre-
Cre+
B) Frequency of CD4+ and CD8+ T cells
% of CD45+
CD4+
CD8+
C)
Cre-
Cre+
77.7
22.3
67.5
32.5
Helios
Foxp3
D) Frequency of CD4+ Foxp3+ T cells
% of CD4+
Foxp3+
E) Frequency of Ki67+ CD4+ T cells
% Ki67 of parent
Foxp3-CD4+
Foxp3+CD4+
F) Phenotypic analysis of CD4+ Foxp3- T cells
gMFI
CD25
CD38
CD39
CD44
Ly6c
CD103
PD-L1
PD1
GITR
ICOS
TIGIT
CTLA4
G) Phenotypic analysis of CD4+ Foxp3+ T cells
H)
99.9
0.031
99.9
0.15
I) Frequency of CD8+ Foxp3+ T cells
% of CD8+
J) Frequency of Ki67+ of CD8+ T cells
Foxp3-CD8+
Foxp3+CD8+
K) Phenotypic analysis of CD8+ Foxp3- T cells
L) Phenotypic analysis of CD8+ Foxp3+ T cells

Figure S4: Immunophenotyping of the T cell landscape in CNS-draining cervical LNs on day 13 after immunization. (A) Schematic representation of the experiment. (B) Frequency of CD4+ and CD8+ T cells. (C) Representative FACS plots showing Helios and Foxp3 expression in CD4+ T cells of Cre- and Cre+ mice. (D) Frequency of CD4+ Foxp3+ Tregs. (E) Frequency of Ki67+ cells among CD4+ Foxp3- and CD4+ Foxp3+ T cells. (F-G) Phenotypic analysis of CD4+ Foxp3- T cells (F) and CD4+ Foxp3+ T cells (G) expressed as geometric mean intensity (gMFI) for the molecules indicated. (H) Representative FACS plots showing Helios and Foxp3 expression in CD8+ T cells of Cre- and Cre+ mice. (I) Frequency of CD8+ Foxp3+ T cells. (J) Frequency of Ki67+ cells among CD8+ Foxp3- and CD8+ Foxp3+ T cells. (K-L) Phenotypic analysis of CD8+ Foxp3- T cells (K) and CD8+ Foxp3+ T cells (L) expressed as geometric mean intensity for the indicated molecules. Graphs represent mean $\pm$ SEM. N = 6 mice / group.
